# Characterization of cancer-related fibroblasts (CAF) in hepatocellular carcinoma and construction of CAF-based risk signature based on single-cell RNA-seq and bulk RNA-seq data

**DOI:** 10.3389/fimmu.2022.1009789

**Published:** 2022-09-23

**Authors:** Lianghe Yu, Ningjia Shen, Yan Shi, Xintong Shi, Xiaohui Fu, Shuang Li, Bin Zhu, Wenlong Yu, Yongjie Zhang

**Affiliations:** ^1^Hepatobiliary Surgery, the third affiliated hospital, Naval Military Medical University, Shanghai, China; ^2^Bioinformatics R&D Department, Hangzhou Mugu Technology Co., Ltd, Hangzhou, China

**Keywords:** cancer-associated fibroblasts, liver hepatocellular carcinoma, differentially expressed genes, immunotherapy, nomogram

## Abstract

**Background:**

Cancer-associated fibroblasts (CAFs) are involved in tumor growth, angiogenesis, metastasis, and resistance to therapy. We sought to explore the CAFs characteristics in hepatocellular carcinoma (HCC) and establish a CAF-based risk signature for predicting the prognosis of HCC patients.

**Methods:**

The signal-cell RNA sequencing (scRNA-seq) data was obtained from the GEO database. Bulk RNA-seq data and microarray data of HCC were obtained from the TCGA and GEO databases respectively. Seurat R package was applied to process scRNA-seq data and identify CAF clusters according to the CAF markers. Differential expression analysis was performed to screen differentially expressed genes (DEGs) between normal and tumor samples in TCGA dataset. Then Pearson correlation analysis was used to determine the DEGs associated with CAF clusters, followed by the univariate Cox regression analysis to identify CAF-related prognostic genes. Lasso regression was implemented to construct a risk signature based on CAF-related prognostic genes. Finally, a nomogram model based on the risk signature and clinicopathological characteristics was developed.

**Results:**

Based on scRNA-seq data, we identified 4 CAF clusters in HCC, 3 of which were associated with prognosis in HCC. A total of 423 genes were identified from 2811 DEGs to be significantly correlated with CAF clusters, and were narrowed down to generate a risk signature with 6 genes. These six genes were primarily connected with 39 pathways, such as angiogenesis, apoptosis, and hypoxia. Meanwhile, the risk signature was significantly associated with stromal and immune scores, as well as some immune cells. Multivariate analysis revealed that risk signature was an independent prognostic factor for HCC, and its value in predicting immunotherapeutic outcomes was confirmed. A novel nomogram integrating the stage and CAF-based risk signature was constructed, which exhibited favorable predictability and reliability in the prognosis prediction of HCC.

**Conclusion:**

CAF-based risk signatures can effectively predict the prognosis of HCC, and comprehensive characterization of the CAF signature of HCC may help to interpret the response of HCC to immunotherapy and provide new strategies for cancer treatment.

## Introduction

Liver cancer is a lethal disease with high prevalence and unfavorable outcomes, where liver hepatocellular carcinoma (HCC) is the primary malignancy of liver cancer, consisting of 75%–85% of cases (1). Although significantly progression in the treatment of HCC, the average 5- year survival rate remains below 20% due to the development of recurrence (2, 3). It has been suggested that several factors such as chronic liver disease, alcohol addiction, and metabolic syndrome, increasing obesity rates, contribute to an increased incidence and mortality of HCC (4). Over the past decades, our understanding of the molecular pathogenesis of HCC has improved significantly thanks to the rapid development of omics technology (5, 6). A series of omic data-derived signatures were generated to predict the clinical outcomes of HCC (7). Accordingly, more novel multigene signature are valuable for predicting the outcome and recurrence of HCC.

The tumor microenvironment (TME) is composed of tumor cells and stromal cells. The malignant potential of tumors has long been thought to be entirely due to cancer cells (8). However, the dynamic crosstalk between cancer cells and stromal cells has been shown to be involved in cancer progression (9). The stroma consists of fibroblasts, pericytes, mesenchymal stem cells, and various types of immune cells, which were surrounded by fibrous structural proteins in the extracellular matrix (10). Cancer-associated fibroblasts (CAFs) are important components of the TME which arise from bone marrow-derived mesenchymal stem cells, hematopoietic stem cells, adipocytes, and endothelial cells (8, 11), as well as cancer cells (12). CAFs have been observed in a majority of cancers, such as breast cancer, prostate cancers, and HCC (13, 14), and its crosstalk with cancer cells has been revealed to be crucial for tumor progression (15). CAFs secrete a variety of growth factors and cytokines, and degrade extracellular matrix proteins, thereby affecting tumor cell proliferation, metastasis and chemotherapy resistance (16–18). CAFs could be stably maintained the tumor-promoting characteristics even without exposure to cancer cells (19). Therefore, it has become a potential strategy to shut down the downstream effects of CAFs or inhibit CAF-secreted factors that facilitate tumor development and progression for HCC intervention.

Although many studies focusing on CAF have been carried out in HCC, the systematic CAF characteristics and its relationship with HCC prognosis and immunotherapy response remain poorly understood. Herein, we obtained HCC single-cell RNA-sequencing (scRNA-seq) data and transcriptome data from accessible databases. We distinguished CAFs subclusters and identified CAF-based risk signature for HCC. Clinical relevance of the CAF-based signature was determined, and the immune landscaoe and responsiveness to immunotherapy underlying the CAF-based signature were further analzyed. Finally, we developed a novel nomogram combining the CAF-based risk signature and clinicopathological features to facilitate the clinical application of CAF features in the prognosis of HCC. It may provide new insights into the pathophysiology of HCC, leading to more tailored treatments and improved outcomes for patients with HCC.

## Materials and methods

### Data acquisition and processing

ScRNA-seq data of GSE149614 was downloaded from the Gene Expression Omnibus (GEO) database, including 10 samples of primary tumors, 2 samples of portal vein tumor thrombi, 1 sample of metastatic lymph node, and 8 samples of non-tumor liver. For scRNA-seq data, single cells were firstly screened with each gene expressing in at least 3 cells and each cell expressing at least 250 genes. Then PercentageFeatureSet function in Seurat R package was conducted to evaluate the proportion of mitochondria and rRNA. The single cells were further screened by setting each cell expressing at least 6000 genes with UMI > 100. Finally, a total of 69145 cells were remained. The transcriptome data, single-nucleotide variant (SNV) and copy number variants (CNV) data of Masked Copy Number Segment, and corresponding clinical information of HCC were obtained from The Cancer Genome Atlas (TCGA) database. For transcriptome data, the samples without survival data and outcome status were removed, and eventually, 360 tumor samples and 50 para-cancerous samples were included. GSE76427 cohort with 115 HCC samples was downloaded from the GEO database as a validation cohort after the removal of normal tissue samples and tumor samples without follow-up and outcome status information. Ten cancer-related pathways (Cell Cycle, HIPPO, MYC, NOTCH, NRF1, PI3K, TGF-Beta, RAS, TP53, and WNT) were retrieved from the literature (20).

### Definition of CAF

We re-analyzed the scRNA-seq data of HCC using the Seurat package (21) to comprehensively characterize the CAF signature. Firstly, we removed the cells with over 6000 or below 250 expressed genes, followed by log normalization of expressed genes. The batch effects for 21 samples were eliminated using the FindIntegrationAnchors function. The non-linear dimensional reduction was conducted using the uniform manifold approximation and projection method, with 15 principal components and a resolution at 0.2. Single cells were clustered into different subgroups by using the functions of FindNeighbors and FindClusters (dim = 40 and resolution = 0.2).Then t-distributed stochastic neighbor embedding (TSNE) dimensional reduction was conducted using the RunTSNE function. Fibroblasts were annotated with 4 marker genes, including ACTA2, FAP, PDGFRB, and NOTCH3. The fibroblasts were re-clustered with the same algorithm of FindNeighbors and FindClusters functions. TSNE dimensionality reduction was further performed on fibroblasts clusters. Marker genes of each CAF cluster were identified using FindAllMarkers function by comparing one cluster with other clusters with logFC = 0.5, minpct = 0.35, and adjusted p-value<0.05. Kyoto Encyclopedia of Genes and Genomes (KEGG) enrichment analysis on the marker genes of CAFs clusters using the clusterProfiler package (22), and the CNV characteristics among the CAFs clusters were analyzed using the CopyKAT R package to differentiate between tumor cells and normal cells in each sample (23).

### Identification of hub genes of CAF

Firstly, the differentially expressed genes (DEGs) between the tumor and normal tissue were screened out *via* limma package with a false discovery rate (FDR)<0.05 and |log2(Fold Change)|>1 (24). Then, we assessed the correlations between the DEGs and CAF clusters, and identify the key CAF-related genes with p<0.001 and cor>0.4. The prognosis-related genes were further identified using univariate Cox regression analysis in survival package with p<0.05 (https://rdocumentation.org/packages/survival/versions/2.42-3). To compress the gene number, we performed the least absolute shrinkage and selection operator (lasso) cox regression analysis, followed by multivariate Cox regression analysis with a stepwise regression method. According to the results of the multivariate Cox model, we constructed a risk signature with the following formula: risk score=Σβi*Expi. Where i is the gene in risk signature, expi represents the expression of the gene i, and βi represents the coefficients of gene i in multivariate Cox model. The patients were divided into the high- and low-risk groups after zero-mean normalization. The receiver operating characteristic curve (ROC) analysis was performed using the timeROC package (https://cran.r-project.org/web/packages/timeROC/index.html) to analyze the predictive performance of the risk signature. Similar analyses were conducted in the validation cohort.

### Immune landscape analysis

The proportions of 22 immune cell subtypes in the TCGA cohort were evaluated by the CIBERSORT algorithm (25), a tool for assessing immune cell infiltration, and the immune and stromal scores were calculated using the ESTIMATE algorithm (https://sourceforge.net/projects/estimateproject/) to further explore the TME.

### Construction of a risk signature and nomogram

To construct a nomogram model for clinical use, we first perform the univariate and multivariate Cox regression analysis on clinicopathological and risk signature. The variables with p<0.05 in the multivariate Cox model were used to construct a nomogram for the prediction of HCC prognosis using the rms package (26). The calibration curve was generated to evaluate the predictive accuracy of the model. The reliability of the model was evaluated using decision curve analysis (DCA).

### Responsiveness to immune checkpoint blocks

We downloaded the transcriptomic, and matched clinical data of patients with HCC treated with an anti-PD-L1 agent (atezolizumab) (27) from IMvigor210 cohort (http://research-pub.gene.com/IMvigor210CoreBiologies). Meanwhile, GSE78220 cohort comprised of transcriptomic data from pre-treatment melanomas receiving anti-PD-1 checkpoint inhibition therapy (28), and also download for the determination of the potential value of the risk signature score in the prediction of responsiveness to immune checkpoint blocks (ICB).

### Statistical analysis

All statistical analyses were performed using the R software (v3.6.3). The correlation matrices were conducted using Pearson or Spearman correlation. Wilcoxon test was conducted for the comparisons between the two groups. Survival differences were compared using K–M curves with a Log-rank test. P-value < 0.05 was considered statistical significance.

## Results

### Screening the CAFs in scRNA-seq samples

**Figure 1:**
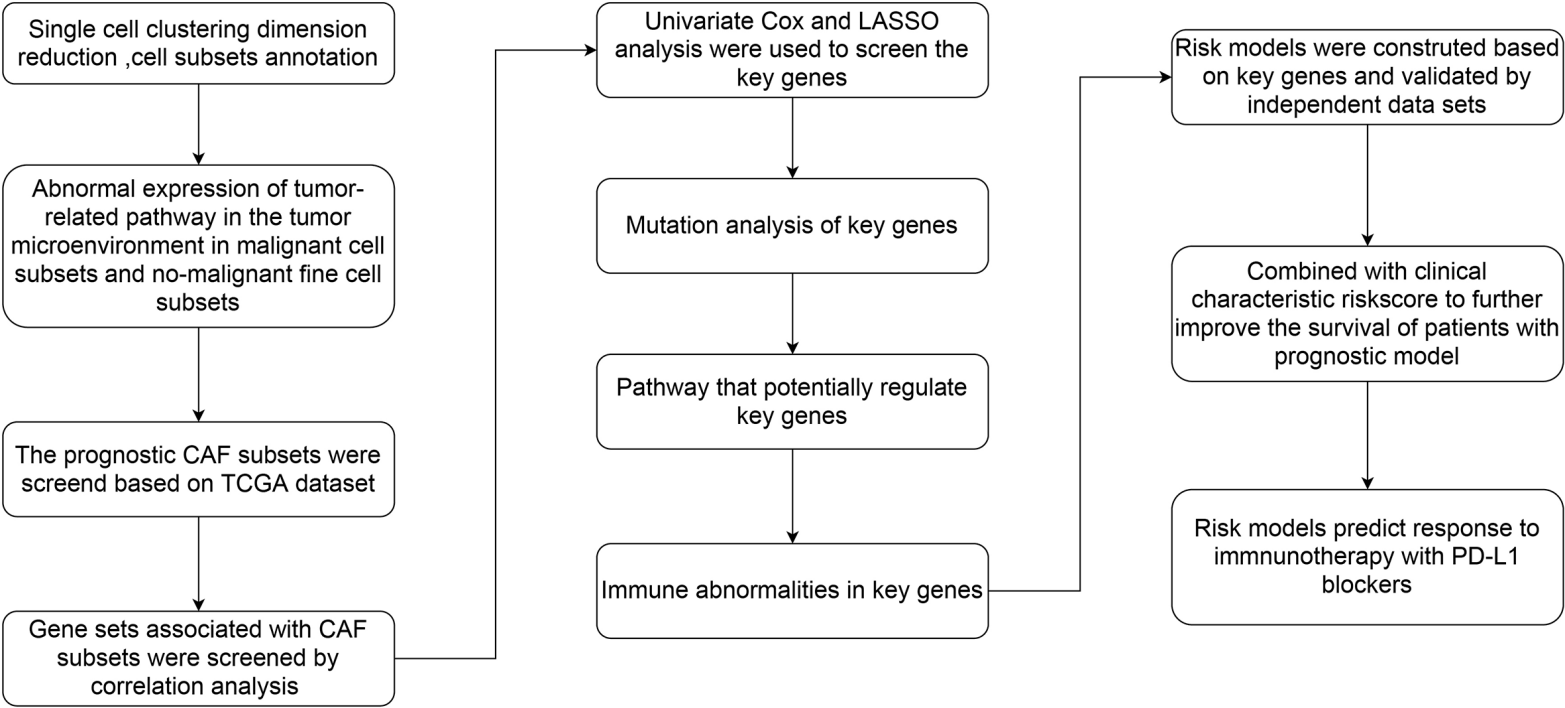
The flow chart of this study.

**Figure 2:**
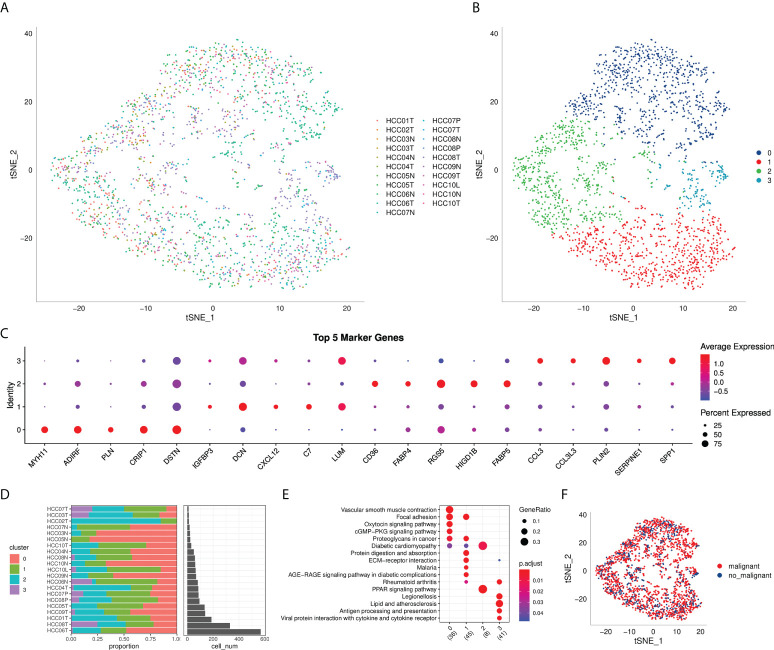
The identification of CAF clusters based on scRNA seq data of HCC patients. **(A)** tsne plot of the distribution of 21 samples; **(B)** tsne plot of the distribution of four fibroblasts after clustering; **(C)** dot plot of the top 5 marker gene expression of subgroups; **(D)** subgroups in cancer tissue and Proportion and cell number of adjacent tissue; **(E)** kegg enrichment analysis of 4 fibroblast subsets; **(F)** tsne distribution map of malignant and non-malignant cells predicted by copykat package.

The flow chart of this study was shown in **Figure 1**. A total of 69145 cells were obtained from the scRNA-seq data after initial screening **(** **Table 1****)**. The detailed results of data preprocessing were shown in **Figure S1**. After log-normalization and dimensionality reduction, 15 subpopulations were obtained, and 9 CAF populations were identified based on four marker genes, including ACTA2, FAP, PDGFRB, and NOTCH3 (**Figures S2A, B**). The cells of 9 CAF populations were extracted for further clustering and dimensionality reduction. The CAF populations were further clustered by using the same clustering algorithm and four CAF clusters were identified (**Figures S2C, D**). The epithelial cell specific gene was not expressed in all four CAF clusters, supporting the accuracy of CAF identification (**Figure S3**). **Figure 2A** showed the TSNE plot of 21 sample distributions. As a result, four CAF clusters were finally generated and used for subsequent analysis (**Figure 2B**). A total of 211 DEGs among the 4 CAF clusters were identified and the expression of the top 5 DEGs (determined as the marker genes of CAF clusters) in the 4 clusters was shown in **Figure 2C**. The proportion of the 4 clusters in each cohort were illustrated in **Figure 2D**. As shown in **Figure 2E**, the results of KEGG analysis demonstrated that these DEGs were enriched in multiple pathways, including vascular smooth muscle contraction, focal adhesion, oxytcosin signaling pathway, PPARG signaling pathway, etc. In addition, the 4 CAF clusters consist of 1533 tumor cells and normal cells according to the CNV characteristics (**Figure 2F**).

**Table 1:** Cell counts before and after filtration of samples.

Samples	raw_count	clean_count	Percentage(%)
HCC01T	3368	3297	97.89
HCC02T	4101	3817	93.07
HCC03N	2601	2601	100
HCC03T	4825	4822	99.94
HCC04N	3396	3380	99.53
HCC04T	3501	2812	80.32
HCC05N	4656	4654	99.96
HCC05T	3353	3250	96.93
HCC06N	4465	4459	99.87
HCC06T	4308	4273	99.19
HCC07N	3740	3739	99.97
HCC07P	1829	1817	99.34
HCC07T	510	507	99.41
HCC08N	4795	4792	99.94
HCC08P	4142	3100	74.84
HCC08T	4833	4657	96.36
HCC09N	1962	1961	99.95
HCC09T	2816	2726	96.8
HCC10L	2843	2742	96.45
HCC10N	3072	3070	99.93
HCC10T	2799	2669	95.36

### The expression of cancer-related pathways in CAF

**Figure 3:**
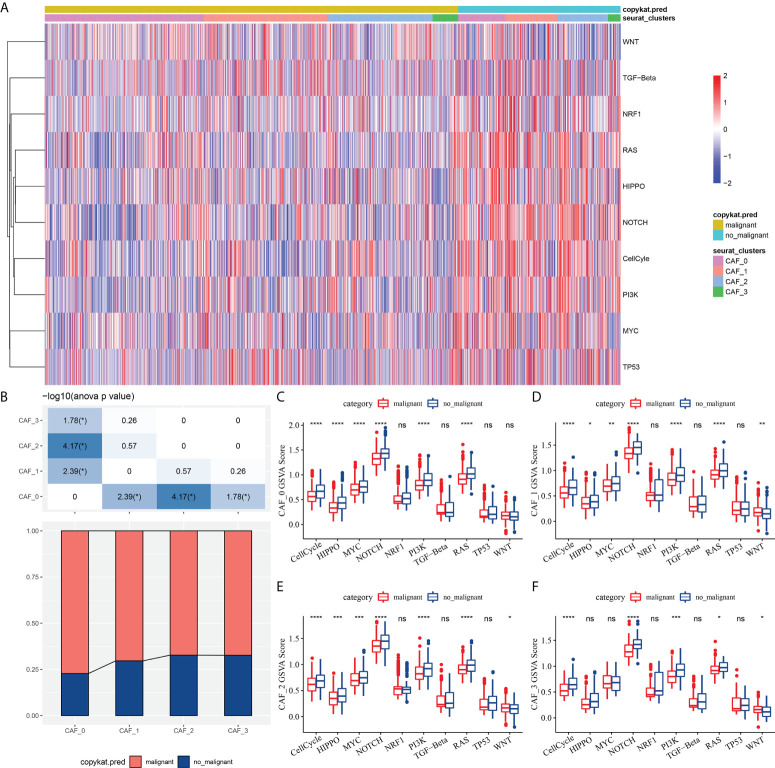
The characteristics of tumor-related pathways in CAF clusters. **(A)** Heatmap of 10 tumor-related pathway scores enriched in CAF cells; **(B)** Comparison of CAF clusters in malignant and non-malignant cells; Comparison of GSVA score of each pathways between malignant and non-malignant cells in CAF_0 **(C)**, CAF_1 **(D)**,CAF_2 **(E)**, and CAF_3 cluster **(F)**. (wilcox.test, *P < 0.05; **P < 0.01; ***P < 0.001; and ****P < 0.0001). ns, not significant.

To elucidate the associations between the CAF clusters and tumor progression, we investigated the characteristics of ten tumor-related pathways in the four CAF clusters. The GSVA scores of the ten tumor-related pathways in different CAF clusters were shown in **Figure 3A**. The ratio of malignant cells in CAF_0 cluster was significantly higher than that in the other three clusters (**Figure 3B**). However, there were no significant differences among the CAF_1, CAF_2, and CAF_3. Furthermore, we analyzed the GSVA scores of the ten tumor-related pathways between malignant and non-malignant cells in each CAF cluster, with slight differences observed (**Figures 3C-F**).

**Figure 4:**
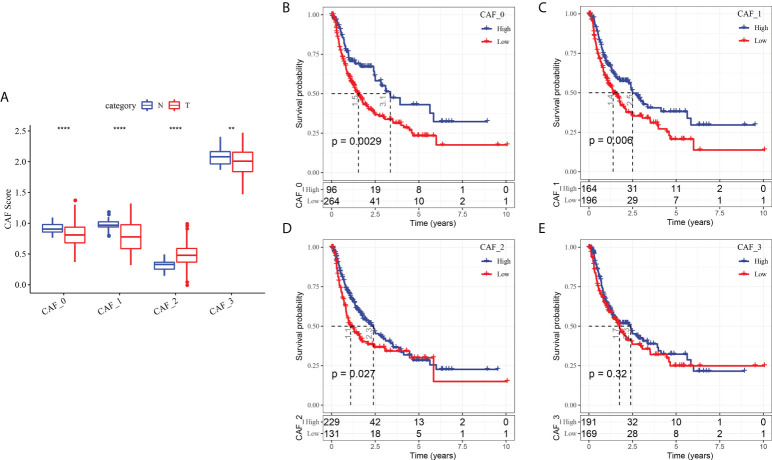
The associations between the four CAF cluster and prognosis of HCC patients. **(A)** Comparison of four CAF scores in cancer and normal tissues; K-M curves of the high and low CAF score groups in the CAF_0 cluster **(B)**, CAF_0 cluster **(C)**, CAF_0 cluster **(D)**, and CAF_0 cluster **(E)**. **P < 0.01, ****P < 0.0001.

To determine the associations between the CAF clusters and prognosis, we first calculated the ssGSEA score of the marker genes (the top 5 DEGs of CAF clusters defined in **Figure 2C**) of each CAF cluster based on the TCGA cohort. The results demonstrated that the CAF_2 cluster had a significantly higher score in tumor samples than in normal samples, whereas the other CAF clusters had an opposite trend, with a higher score in normal samples than in tumor samples (**Figure 4A**). The HCC samples of TCGA dataset were separated into the high- and low-CAF score groups according to the optimal cut-off value analyzed by survminer R package. The samples in the high-CAF score group had a better prognosis in the CAF_0, CAF_1, and CAF_2 clusters than those in the low-CAF score group, whereas the CAF_3 was not associated with the prognosis of HCC (**Figures 4B-E**). The above results suggested that CAF_3 cluster may contribute little in the HCC progression although CAF_3 enrichment was differential in HCC and normal samples.

### Identification of hub genes associated with CAF

**Figure 5:**
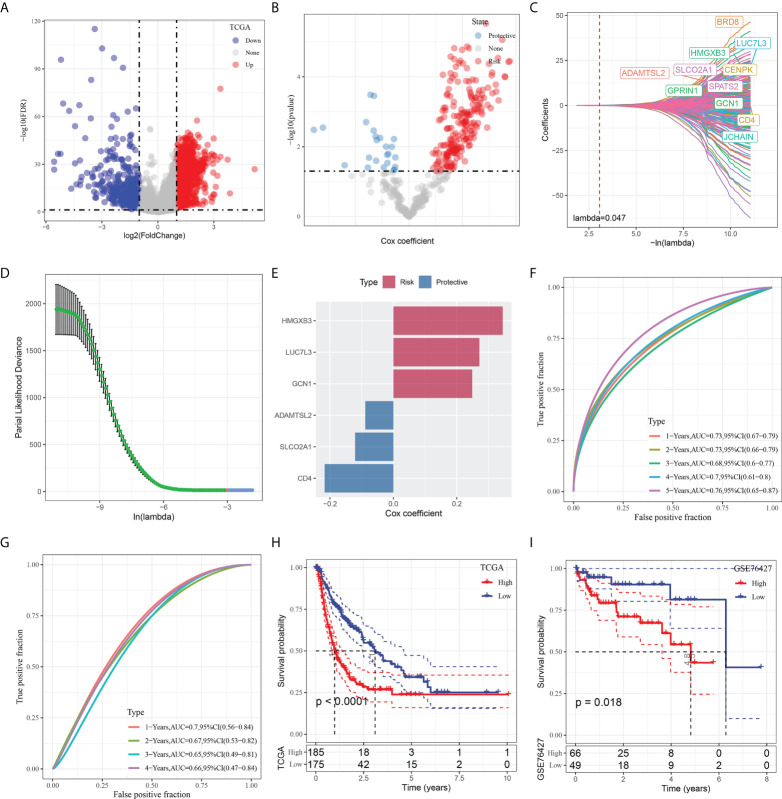
Identification of the hub predictive genes to construct a risk signature. **(A)** Volcano plot of differentially expressed genes of cancer and normal tissues in TCGA cohort; **(B)** Volcano plot of prognosis-related genes identified from univariate Cox regression analysis; **(C)** The trajectory of each independent variable with lambda; **(D)** Plots of the produced coefficient distributions for the logarithmic (lambda) series for parameter selection (lambda); **(E)** The multivariate Cox coefficients for each genes in the risk signature. **(F)** and **(G)** ROC curves of risk model constructed by 6 genes in TCGA cohort and GEO cohort; **(H)** and **(I)** K-M curves of risk model constructed by 6 genes in TCGA cohort and GEO cohort.

To construct a risk signature, we firstly screened out DEGs between the tumor and normal tissues. As shown in **Figure 5A**, a total of 2349 DEGs were obtained, with 462 up-regulated DEGs and 1887 down-regulated DEGs. Among them, there are 423 genes that showed significant correlations with those prognosis-related CAF clusters. Furthermore, the prognostic value of each gene was assessed *via* univariate Cox regression analysis, with 234 genes exhibiting prognostic values (**Figures 5A, B**). Lasso Cox regression analysis was performed to narrow down the number of genes, with 11 genes left as lambda=0.047 (**Figures 5C, D**). Finally, we included 6 genes, including HMG-box containing 3 (HMGXB3), GCN1 activator of EIF2AK4 (GCN1), LUC7 like 3 pre-mRNA splicing factor (LUC7L3), ADAMTS like 2 (ADAMTSL2), solute carrier organic anion transporter family member 2A1 (SLCO2A1), and CD4 molecule (CD4), in the risk signature after multivariate Cox regression analysis with stepwise regression method (**Figure 5E**). The final 6-gene signature formula is as follows: RiskScore = -0.088*ADAMTSL2 - 0.121*SLCO2A1 - 0.217*CD4 + 0.249*GCN1 + 0.345*HMGXB3 + 0.271*LUC7L3. We calculated the risk score for each sample and divided them into the high- and low-risk groups after z-mean normalization. The AUC values of the model for 1- to 5-year survival range from 0.68 to 0.76 in the TCGA cohort and range from 0.65 to 0.7 in the GEO cohort (**Figures 5F, G**). Kaplan-Meier survival analyses revealed that high-risk patients had significantly poorer survival outcomes compared with low-risk patients in the TCGA cohort, as well as in the GEO cohort (**Figures 5H, I**).

### Mutation and pathway analysis of the hub genes

**Figure 6:**
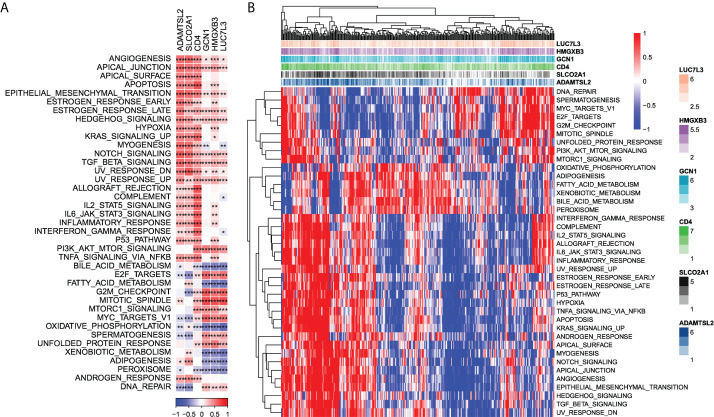
Identification of pathways that the risk genes involved in. **(A)** Gene-pathway correlation heatmap; **(B)** Enrichment score heatmap for key pathways. *P < 0.05, **P < 0.01, ***P < 0.001.

Next, we checked out the SNV mutations of the six genes of the risk signature. It showed that ADAMTSL2, SLCO2A1, HMGXB3, LUC7L3, and CD4 have SNV mutations in more samples, while no SNV mutation was observed in GCN1 (**Figure S4A**). We analyzed the co-occurrence probability of these key genes and the 10 most mutated genes. As revealed in **Figure S4B**, there was no significant probability of co-occurrence of the mutations in these 5 genes, but LUC7L3 presented a significant probability of co-occurrence with ABCA13 mutation. In the 6 genes, it was found that only a very small number of samples had gain/loss of CNV (**Figure S4C**). To further elucidate the associations between the risk genes and HCC, we analyzed the correlations between these genes and several molecular signatures of HCC. The results demonstrated that SLCO2A1 had significantly negative correlations with Aneuploidy Score, Homologous Recombination Defects, Fraction Altered, Number of Segments, and Nonsilent Mutation Rate, whereas HMGXB3, LUC7L3, and GCN1 showed significantly positive correlations with Homologous Recombination Defects and Fraction Altered (**Figure S4D****)**. In addition, we analyzed the potential pathways associated with each risk gene. As shown in **Figures 6A, B**, a total of 39 pathways were significantly correlated with these six genes, including angiogenesis, apical junction, apoptosis, etc.

### Relationship between hub genes and immunity

Our data demonstrated that ADAMTSL2, SLCO2A1, and CD4 presented significantly positive correlations with the stromal score, immune score, and estimate score, while LUC7L3 showed significantly negative correlations with the stromal, immune, and estimate scores. However, there was no significant correlations observed between the three scores and the other genes (GCN1 and HMGXB3) (**Figure S5A**). After grouping according to the median value of expression of each gene, we compared the three scores in different expressed groups. The results showed that, with regard to the ADAMTSL2, SLCO2A1, and CD4 genes, the three scores of the high expression group were significantly higher than those of the low expression group (**Figure S5B**). Correlation analysis revealed that ADAMTSL2, SLCO2A1, and CD4 presented a significantly negative correlation with the majority of T cells. Additionally, LUC7L3, GCN1, and HMGXB3 significantly positively correlated with M0 macrophages and neutrophils (**Figure S5C**). Moreover, we also observed significant differences between the high and low expression groups of risk genes in several immune cells (**Figure S5D**).

### The responsiveness of risk signature to PD-L1 blockade immunotherapy

**Figure 7:**
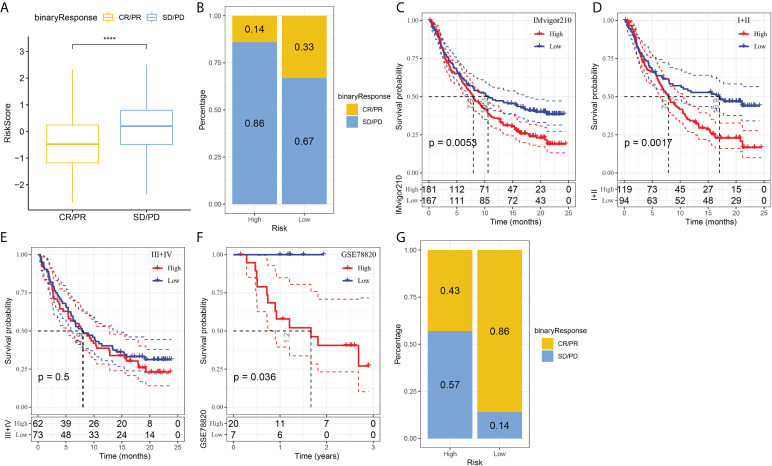
The responsiveness of risk score to PD-L1 blockade immunotherapy in IMvigor210 cohort. **(A)** Differences in risk scores among immunotherapy responses in the IMvigor210 cohort; **(B)** Distribution of immunotherapy responses among risk score groups in the IMvigor210 cohort; **(C)** Prognostic differences among risk score groups in the IMvigor210 cohort; **(D)** Prognostic differences between risk score groups in early stage patients in the IMvigor210 cohort; **(E)** prognostic differences between risk score groups in advanced patients in the IMvigor210 cohort; **(F)** prognostic differences in risk score groups in the GSE78220 cohort; **(G)** Distribution of immunotherapy responses among risk score groups in the GSE78220 cohort. ****P < 0.0001.

T-cell immunotherapy has emerged as an anticancer treatment with synergistic survival benefits (29). Therefore, we assessed the prognostic value of risk signature for immune-checkpoint therapy in the IMvigor210 and GSE78220 cohorts. The 348 patients in the IMvigor210 cohort showed varying degrees of response to anti-PD-L1 receptor blockers, including complete response (CR), partial response (PR), stable disease (SD), and progressive disease (PD). SD/PD patients presented higher risk scores than CR/PR patients (**Figure 7A**). In the high-risk group, the percentage of SD/PD was higher than that in the low-risk group (**Figure 7B**). We observed that in the IMvigor210 cohort, patients in the low-risk group showed significant clinical benefits and a significantly longer overall survival as compared with those in the high-risk group (**Figure 7C**, p=0.0053). Specifically, there were significant survival differences in Stage I+II patients between the different risk groups (**Figure 7D**, p=0.0017), but not in Stage III+IV patients (**Figure 7E**, p=0.5). It suggested that the risk score is more sensitive in early-stage patients. In the GSE78220 cohort, we also found a significantly longer overall survival of patients in low-risk group than in high-risk group (**Figure 7F**, p=0.036). Meanwhile, the percentage of SD/PD in the high-risk group was higher than that in the low risk group (**Figure 7G**).

### Identification of independent risk factors and nomogram developing

**Figure 8:**
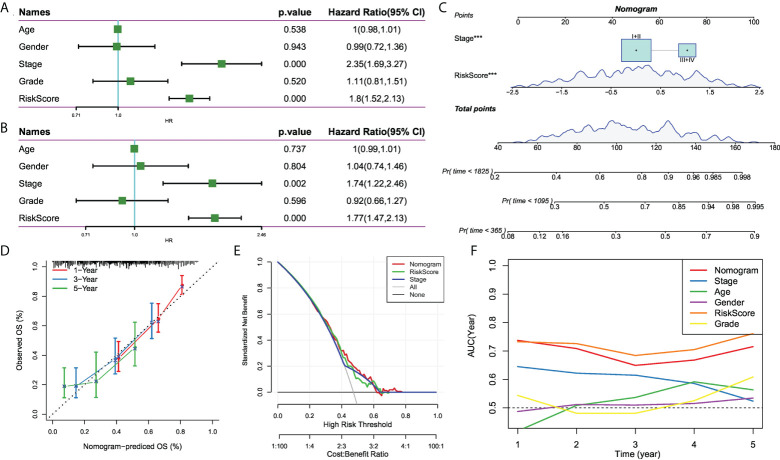
The development of a nomogram for predicting the prognosis of HCC. **(A, B)** Univariate and multivariate Cox analysis of risk score and clinicopathological characteristics; **(C)** Nomogram model integrating the risk score and stage was constructed; **(D)** Calibration curves for 1, 3, and 5 years of nomogram; **(E)** Decision curve for nomogram; **(F)** Comparison of predictive capacity of clinicopathological features and the nomogram using time-ROC analysis. ***P < 0.001

To optimize the predictive performance of the risk signature, we integrated the clinicopathological characteristics and risk score *via* univariate and multivariate Cox regression analysis. Multivariate analysis demonstrated that risk signature was the most significant independent prognostic factor of osteosarcoma [hazard ratio (HR) = 1.77, 95% confidence interval (CI): 1.42 - 2.13, P < 0.001], followed by metastatic status (HR = 1.74, 95%CI: 1.22 - 2.46, P = 0.002) (**Figures 8A, B**). Therefore, a nomogram combining stage and risk score was constructed, as shown in **Figure 8C**. The calibration plot demonstrated that the nomogram can effectively forecast the actual survival outcomes (**Figure 8D**). Moreover, DCA revealed a better discriminative ability of the nomogram in recognizing patients at high risk than the risk score and stage, as shown in **Figure 8E**. TimeROC analysis showed that the AUC of the risk score and nomogram was higher than that of other indicators in the TCGA cohort (**Figure 8F**).

## Discussion

Accumulating evidence has demonstrated the dynamic crosstalk between tumor cells and the stromal cells contributes to tumor progression (9), As CAFs have been confirmed to be involved in tumor proliferation, angiogenesis, metastasis, as well as chemotherapy resistance *via* releasing various factors into the TME (30). In the present study, we concentrated on the diversity of CAFs and performed a systematic characterization and classification of CAFs of HCC based on scRNA-seq data. Eventually, we identified four CAF clusters with distinct properties, which might contribute to the regulation of different aspects of the biology of the TME. Accumulating evidence has confirmed the prognostic value of CAF-secreted factors or CAF-related gene signature in HCC (31). Consistently, our data revealed three of the clusters with a significant association with HCC prognosis, which were determined using a score derived from DEGs across the four clusters. Interestingly, we observed the differences in HIPPO and MYC among the CAF clusters might may partially contribute to the prognostic value of CAF. Hepatic Hippo signaling inhibits development of HCC (32), and the MYC-mediated axis has been confirmed as a dominant part of HCC in terms of proliferation, migration, invasion, and drug resistance (33).

Given the prognostic values of three CAF clusters, we established a CAF-based risk signature with 6 genes. It consisted of three protective genes (ADAMTSL2, SLCO2A1, and CD4) and three risk genes (HMGXB3, GCN1, and LUC7L3). In our study, SNV mutations were observed in ADAMTSL2, SLCO2A1, HMGXB3, LUC7L3, and CD4 without significant co-occurrence probability. Sense SNV mutations affect protein activity or function, leading to HCC development or affecting HCC progression (34). Although there are no independent studies linking SNV mutations in these risk genes to HCC progression, our data also suggest a potential role of SNV mutations in these genes in HCC progression. A recent study constructed a genomic instability-derived genes signature, which contains SLCO2A1, for the prediction of HCC prognosis (35). We further found that the six genes were significantly correlated with 39 pathways, while protective genes and risk genes clearly had different pathway signatures. For example, the protective genes were significantly positively associated with allograft rejection, myogenesis, complement, interferon-gamma response, whereas risk genes were significantly connected with fatty acid metabolism, xenobiotic metabolism, and adipogenesis. The alterations of fatty acid metabolism plays an important role in HCC and the prognostic value of fatty acid metabolism-related genes in HCC has also been revealed (36). Polymorphisms in xenobiotic metabolism-related genes were suggested to increase the risk of developing HCC (37). Adipogenesis is an indication of the development of obesity and is associated with multiple cancers (38). Hence, these data provide us with the direction to further study the regulation of these risk genes in HCC.

Recent evidence suggests that the interaction of CAFs and the tumor immune microenvironment (TIME) can promote tumor progression (39). In our study, three predictive genes were significantly positively correlated with immune score, while a risk gene was negatively associated with the immune score. These data indicated the potential crosstalk between these genes with TIME in HCC and implied the potential values of these genes as therapeutic targets of HCC. Various immune cells in tumor islets make up the TIME and synergistically determine the antitumor immunological state in the TME. CAFs can interact with these immune cells to form a immunosuppressive TME, thereby enable tumor cells to evade the surveillance of the immune system (40). In the risk signature, multiple types of T cells were negatively associated with the predictive genes. T cells are involved in tumor progression and the potential of T cell-derived therapies, including checkpoint blockade and chimeric antigen receptors T (CAR-T) cell therapy, has been confirmed (41).

Nevertheless, most of the patients show innate or acquired resistance to immunotherapies (42). Our data found that the risk signature was capable to distinguish patients who were more likely to benefit from immunotherapies. Additionally, it was reported that CAF-expressed endosialin regulated macrophage recruitment and polarization in HCC (43). In the defined signature, the risk genes were positively correlated with M0 macrophages and negatively correlated with M2 macrophages, indicating the potential involvement of the risk genes in the macrophage polarization. It was demonstrated that CAFs regulated neutrophil survival, activation, and function in HCC *via* the IL6-STAT3-PDL1 signaling cascade (44). Meanwhile, our data showed that CAF-based signature could predict the responsiveness to anti-PD-L1 immunotherapy. There data provided novel clues of the role of CAF in remodeling the cancer niche and immune status in TME. However, it requires more experiments to explore the role of CAF-TIME communication in HCC and its potential value in HCC immunotherapy.

Nevertheless, several limitations in our study should be acknowledged. First, the CAF clusters and CAF-based risk signature was generated using retrospective data from public databases. Therefore, it should be validated in more prospective and multi-center HCC cohorts in the future. Second, we only investigated the potential prognostic value of the CAF-based risk signature, so further studies are required to explore the underlying mechanisms of the signature in the development of HCC.

## Conclusion

In summary, this study systematically characterized the CAF populations in HCC and generated four CAF clusters with distinct diversity. The DEGs among the four clusters were enriched in vascular smooth muscle contraction, focal adhesion, oxytcosin and PPARG signaling pathway, etc. Three of the cluster were significantly associated with HCC prognosis, and used to construct a CAF-based prognostic risk signature with 6 genes. The CAF-based gene signature was observed to be connected with the immune landscape and could be used for the prediction of the responsiveness to PD-L1 blockade immunotherapy. Finally, a novel nomogram integrating the risk signature and clinicopathological features were developed, which provided a favorable predictive performance in the clinical outcome of patients with HCC.

## Data availability statement

The original contributions presented in the study are included in the article/**Supplementary Material**. Further inquiries can be directed to the corresponding authors.

## Author contributions

LY and NS conducted statistical analyses of the data and prepared the draft manuscript. YS, XS and XF edited the manuscript. SL, BZ, WY and YZ provide critical comments to the manuscript. All authors checked and proofread the final version of the manuscript.

## Funding

This study was supported by Sangerbox platform (45) and Shanghai Science and Technology Committee (09411965400).

## Conflict of interest

Author SL is employed by Bioinformatics R&D Department, Hangzhou Mugu Technology Co., Ltd.

The remaining authors declare that the research was conducted in the absence of any commercial or financial relationships that could be construed as a potential conflict of interest.

## Publisher’s note

All claims expressed in this article are solely those of the authors and do not necessarily represent those of their affiliated organizations, or those of the publisher, the editors and the reviewers. Any product that may be evaluated in this article, or claim that may be made by its manufacturer, is not guaranteed or endorsed by the publisher.

## Supplementary material

Supplementary Figure 1 The results of re-process of scRNA-seq data of LIHC. **(A)**: The relationship between mitochondrial genes and the amount of UMI/mRNA, the relationship between UMI and the amount of mRNA; **(B)** The relationship among mRNA, UMI, mitochondrial content, and rRNA content of each sample before filtering; **(C)** The relationship among mRNA, UMI, mitochondrial content, and rRNA content of each sample after filtering; **(D)** The sample distribution map of PCA dimensionality reduction and the anchor point map of PCA.

Supplementary Figure 2 The clustering of CAF populations and dimensionality reduction. **(A)** Distribution of subpopulations after clustering of all cells; **(B)** TSNE map of fibroblast marker gene expression; **(C)** Distribution of subpopulations after re-clustering of fibroblasts; **(D)** TSNE diagram.of marker expression in four CAF clusters.

Supplementary Figure 3 The expression of EPCAM in four CAF clusters.

Supplementary Figure 4 The characteristics of mutations of the genes included in the risk signature. **(A)** Waterfall diagram of SNV mutations of 6 key genes; **(B)** Colinearity and mutual exclusion analysis of key genes and the 10 most mutated genes in tumors; **(C)** CNV mutations (gain, loss, none) of 6 key genes; **(D)** Correlation heatmap of 6 key genes with Aneuploidy Score, Homologous Recombination Defects, Fraction Altered, Number of Segments, and Nonsilent Mutation Rate.

Supplementary Figure 5 The relationship between the risk genes and immune landscape. **(A)** The correlation matric of the risk genes and stromal score, immune score, and estimate score. **(B)** Comparison of high and low expression of key genes and immune score (wilcox.test); **(C)** Correlation between key genes and immune cell score predicted by CIBERSORT analysis; **(D)** Comparison of high and low expression of key genes with 22 immune cell scores (wilcox.test). *P < 0.05; **P < 0.01; ***P < 0.001; and ****P < 0.0001.
