# Supplementary figures and images for: Characterization of cancer-related fibroblasts (CAF) in hepatocellular carcinoma and construction of CAF-based risk signature based on single-cell RNA-seq and bulk RNA-seq data
Source: Front Immunol. 2022 Sep 23;13:1009789. doi: 10.3389/fimmu.2022.1009789 (PMC9537943; doi:10.3389/fimmu.2022.1009789)

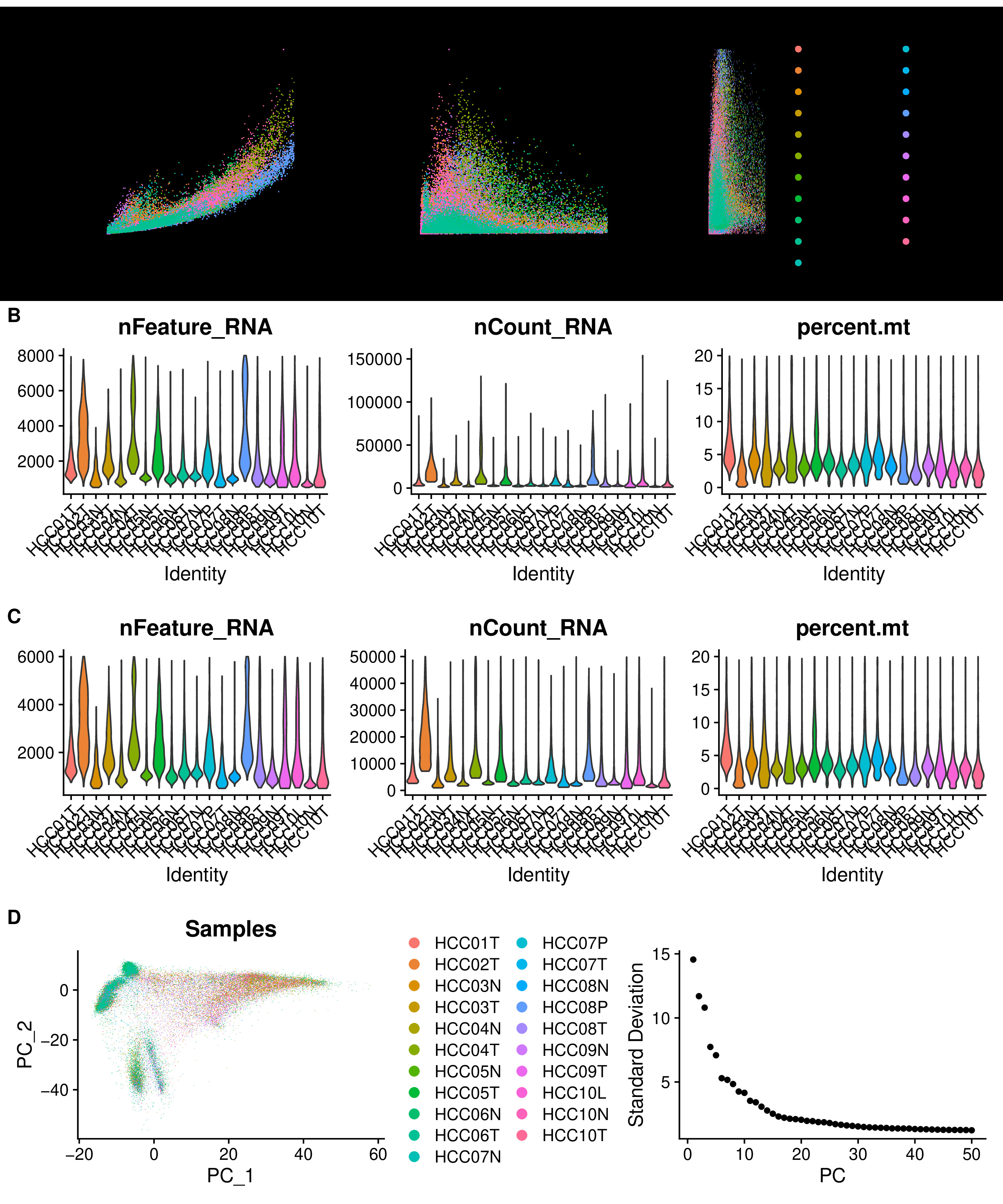

Supplement: Supplementary Figure 1 — The results of re-process of scRNA-seq data of LIHC. (A): The relationship between mitochondrial genes and the amount of UMI/mRNA, the relationship between UMI and the amount of mRNA; (B) The relationship among mRNA, UMI, mitochondrial content, and rRNA content of each sample before filtering; (C) The relationship among mRNA, UMI, mitochondrial content, and rRNA content of each sample after filtering; (D) The sample distribution map of PCA dimensionality reduction and the anchor point map of PCA. [file Image_1.jpeg]

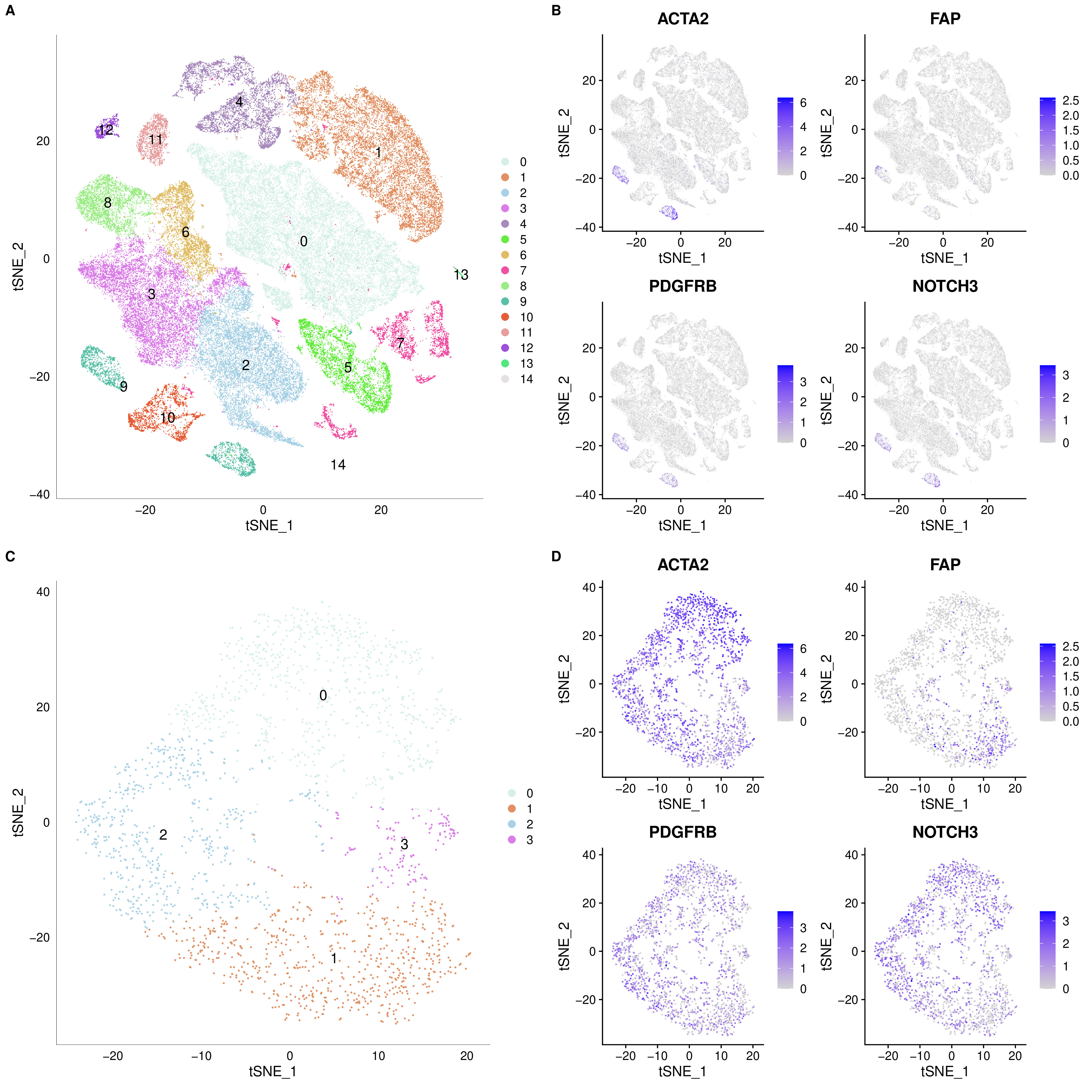

Supplement: Supplementary Figure 2 — The clustering of CAF populations and dimensionality reduction. (A) Distribution of subpopulations after clustering of all cells; (B) TSNE map of fibroblast marker gene expression; (C) Distribution of subpopulations after re-clustering of fibroblasts; (D) TSNE diagram.of marker expression in four CAF clusters. [file Image_2.jpeg]

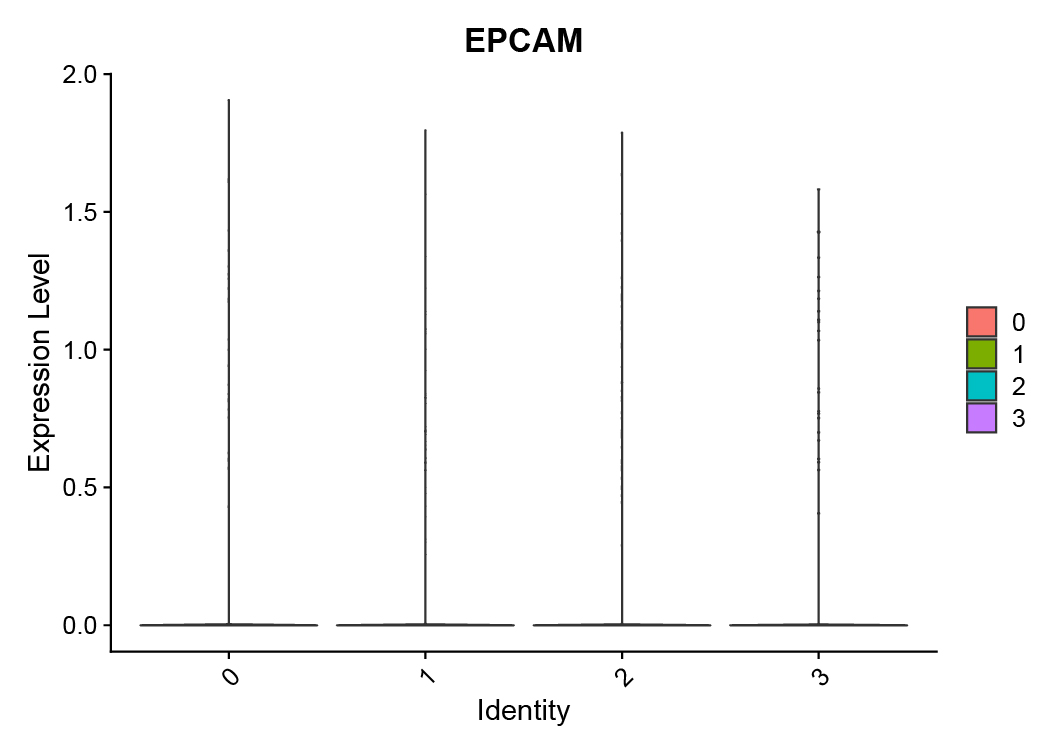

Supplement: Supplementary Figure 3 — The expression of EPCAM in four CAF clusters. [file Image_3.jpeg]

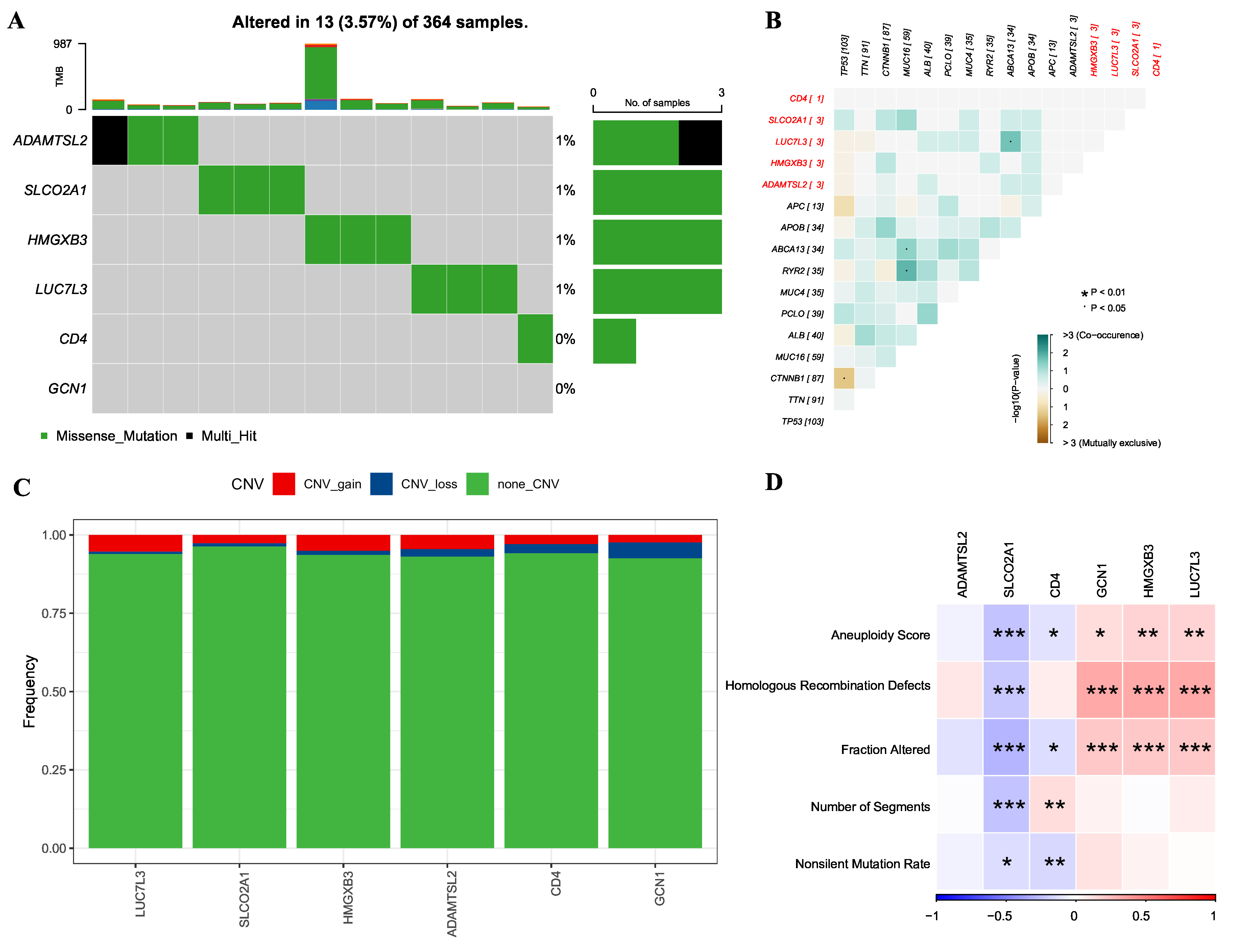

Supplement: Supplementary Figure 4 — The characteristics of mutations of the genes included in the risk signature. (A) Waterfall diagram of SNV mutations of 6 key genes; (B) Colinearity and mutual exclusion analysis of key genes and the 10 most mutated genes in tumors; (C) CNV mutations (gain, loss, none) of 6 key genes; (D) Correlation heatmap of 6 key genes with Aneuploidy Score, Homologous Recombination Defects, Fraction Altered, Number of Segments, and Nonsilent Mutation Rate. [file Image_4.jpeg]

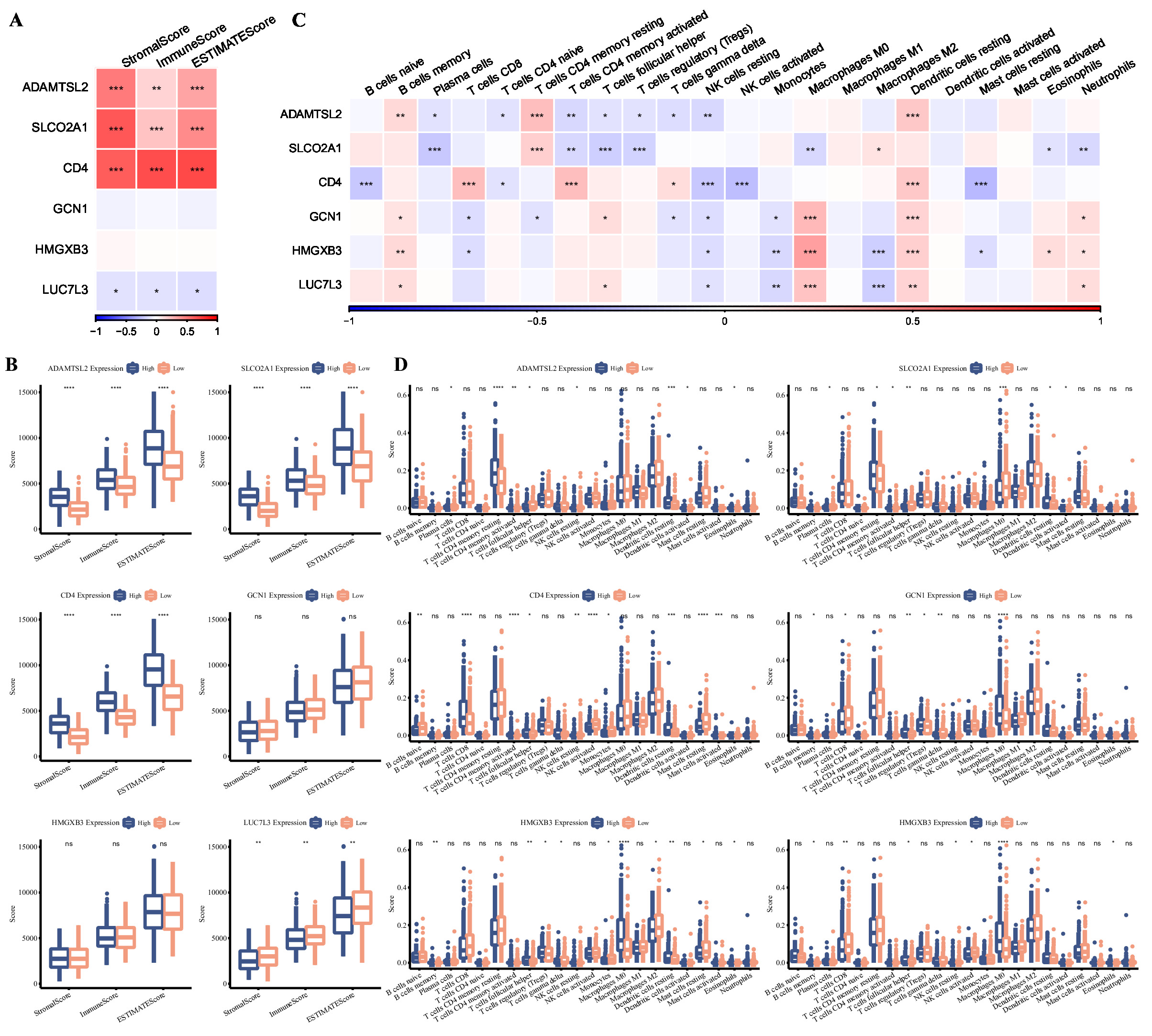

Supplement: Supplementary Figure 5 — The relationship between the risk genes and immune landscape. (A) The correlation matric of the risk genes and stromal score, immune score, and estimate score. (B) Comparison of high and low expression of key genes and immune score (wilcox.test); (C) Correlation between key genes and immune cell score predicted by CIBERSORT analysis; (D) Comparison of high and low expression of key genes with 22 immune cell scores (wilcox.test). *P < 0.05; **P < 0.01; ***P < 0.001; and ****P < 0.0001. [file Image_5.jpeg]
